# Evaluation of Singh Index and Osteoporosis Self-Assessment Tool for Asians as risk assessment tools of hip fracture in patients with type 2 diabetes mellitus

**DOI:** 10.1186/s13018-017-0539-6

**Published:** 2017-03-03

**Authors:** Zhenyu Liu, Hua Gao, Xiaodong Bai, Liang Zhao, Yadong Li, Baojun Wang

**Affiliations:** Department of Orthopaedics, Beijing Friendship Hospital, Capital Medical University, Yongan Road No.95, Xicheng District, 100050 Beijing, China

**Keywords:** Hip fracture, Self-Assessment Tool for Asians, Singh Index, Type 2 diabetes mellitus

## Abstract

**Background:**

Type 2 diabetes mellitus (T2DM), an epidemic disease around world, has recently been identified as a risk factor for osteoporosis-associated fracture. However, there is no consensus on the best method of assessing fracture risk in patients with T2DM. The aim of this study was to evaluate the usefulness of the Osteoporosis Self-Assessment Tool for Asians (OSTA) and the Singh Index (SI) in hip fracture risk assessment in patients with T2DM.

**Methods:**

We enrolled 261 postmenopausal women with T2DM: 87 had hip fracture resulting from low-energy trauma and 174 age-matched controls had no fracture (two controls per fracture case). Bone mineral density (BMD) was measured with dual-energy X-ray absorptiometry in the lumbar spine and hip region. The SI was obtained from standard antero-posterior radiographs of the pelvis. The OSTA was calculated with a formula based on weight and age. Data were analyzed with descriptive statistics and tests of difference. Receiver operating characteristic analysis was used to determine optimum cutoff values, sensitivity, and specificity of screening methods. Discriminative abilities of different screening tools were compared with the area under the curve (AUC).

**Results:**

There were significant differences in BMD at all sites (lumbar spine, femoral neck, trochanter, and total hip) and in SI between the fracture and non-fracture groups (*P* < 0.05). There was no significant difference in OSTA between the groups (*P* > 0.05). The area under the curve was 0.747 (95% CI: 0.680–0.813) for lumbar spine BMD, 0.699 (95% CI: 0.633–0.764) for total hip BMD, 0.659 (95% CI: 0.589–0.729) for femoral neck BMD, 0.631 (95% CI: 0.557–0.704) for trochanter BMD, 0.534 (95% CI: 0.459–0.610) for OSTA, 0.636 (95% CI: 0.564–0.709) for SI, and 0.795 (95% CI: 0.734–0.857) for OSTA plus SI. The AUC for combined OSTA plus SI was significantly superior to other parameters besides BMD of the lumbar spine.

**Conclusions:**

The combination of OSTA plus SI could be a clinical alternative tool for screening of hip fracture risk in large diabetic populations. These tests are inexpensive and simple to perform and could be especially useful in areas where BMD measurement is not accessible.

## Background

Diabetes mellitus is an epidemic disease associated with substantial comorbidity. The number of people with type 2 diabetes mellitus (T2DM) is expected to steadily increase worldwide [1]. T2DM has recently been identified as an important risk factor for osteoporosis-associated fracture, depending on skeletal site and disease severity [2–4]. Bone mineral density (BMD) by dual-energy X-ray absorptiometry (DXA) is recognized as a major tool to detect osteoporosis and predict fracture risk. However, because of the complex pathophysiology of T2DM, BMD studies in diabetic patients have shown contradictory results, including normal, reduced, and increased BMD [5–8]. As a result of this controversy, there is no consensus on the best method of assessing fracture risk in patients with T2DM [9–11].

The Singh Index (SI) is a simple, semiquantitative evaluation tool for diagnosing osteoporosis with plain radiographs [12]. The SI is based on the trabecular pattern of the proximal femur and classifies osteoporosis into six grades. This method is available for routine use and mass screening because plain films can be obtained at most outpatient clinics. Several studies have confirmed that the SI is an effective tool to assess proximal femoral bone strength [13–15]. However, the reliability and accuracy of the SI remain controversial compared with BMD assessment. In addition, the SI was proposed to contain independent information about osteoporosis, which might reflect structural integrity in trabecular bone [13, 16, 17]. Several studies have suggested that a combination of reduced bone mass and altered bone quality, which are not assessed with BMD alone, are important risk factors for fracture in diabetic patients [18, 19]. Therefore, SI may be used as a screening tool to help clinicians identify patients at increased fracture risk.

The Osteoporosis Self-Assessment Tool for Asians (OSTA) has been developed to discriminate patients with high osteoporosis risk for BMD measurement by using a formula [20, 21]. In previous studies, the OSTA has been shown to effectively predict osteoporosis risk and to determine appropriate use of BMD testing in Asian countries without sufficient DXA equipment [22–25]. A few studies have also demonstrated that OSTA may help to identify fracture risk among postmenopausal women [26–28]. Based on a study in eight Asian countries, the OSTA index uses only age and weight from among 11 risk factors to classify the risk of osteoporosis [29]. Because T2DM is associated with increased body weight, the OSTA may underestimate fracture risk in diabetic patients and its validity in these patients remains unknown.

With current demographic changes, physicians will need to treat an increasing number of diabetic patients at a higher risk of osteoporosis. Hip fracture is the most devastating consequence of osteoporosis, with high morbidity, mortality, and economic burden. However, DXA scans are not available to evaluate bone health at all healthcare centers because this diagnostic method is expensive and complicated. Thus, clinicians need a simple and inexpensive screening method to discriminate those patients at high risk of hip fracture from among the large number of patients with diabetes. In the present analysis, we investigated the discriminative ability of OSTA and SI alone and in combination in predicting hip fracture risk in T2DM patients. We sought an alternative method of identifying those diabetic patients at risk for hip fracture in daily clinical practice.

## Methods

This retrospective study was conducted at a single university teaching center. Postmenopausal women with T2DM and femoral neck or intertrochanteric fracture resulting from low-energy trauma were retrospectively recruited from 2009 to 2015. Patients who had undergone BMD measurement of the spine and of the hip contralateral to the fracture site at the time of fracture were eligible for inclusion. Control cases were selected from our pool of patients with BMD testing results and medical records. For every patient with hip fracture, we included two matched controls with T2DM, age ± 2 years, and no low-energy fracture. Exclusion criteria for all participants were the following: (1) any treatment or illness that would affect bone metabolism, (2) history of any spinal surgery, and (3) severe scoliosis or degenerative lumbar disease that would affect BMD measurement.

In all cases, T2DM was diagnosed on the basis of medical records and questionnaire. If a diabetes diagnosis was made during the 5 years prior to BMD testing, the T2DM status was confirmed. Body mass index (BMI) was calculated with the following standard formula: BMI = weight (kg)/height (m)^2^.

### BMD measurements

BMD testing was performed with a single DXA scanner (Discovery W; Hologic, Inc., Bedford, MA, USA). An anatomical spine phantom was measured daily for quality control at our institution. The coefficient of variation of the technique was 0.8%, indicating stable results. Every participant had BMD measured at the left hip and at the lumbar spine from L1 to L4. In patients with hip fracture, BMD was measured on the contralateral hip. All BMD measurements were made by two experienced technicians. The scanning procedures and analysis were performed according to the standard manual supplied by the manufacturer. BMD was calculated on the basis of normal reference values for the Chinese population, in accordance with manufacturer recommendations. *T*-scores were used to express BMD. BMD was classified based on the World Health Organization criteria: *T*-score ≤−2.5 SD, osteoporotic; *T*-score >−2.5 and <−1.0 SD, osteopenic; *T*-score ≥−1.0, normal.

### OSTA calculation

The OSTA index was calculated with the following formula: [body weight (kg) − age (years)] × 0.2. Digits after the decimal point were disregarded. The OSTA values were classified as follows: <−4, high risk; −4 to −1, intermediate risk; >−1, low risk.

### Evaluation of the Singh Index

Standard digital antero-posterior radiographs of the pelvis in the supine position were obtained to assess the SI. According to the Singh criteria, SI scores ranged from 1 (severe osteoporosis) to 6 (normal bone density). SI was assessed in the proximal femur on the same side used for BMD measurement. All radiographs were evaluated by a single observer.

The following parameters were collected for each participant: patient age, weight, height, BMI, fracture site, BMD, OSTA, and SI.

### Statistical analysis

Statistical analyses were performed with SSPS software 18.0 (SPSS, Chicago, IL, USA). Descriptive statistics are expressed as mean ± standard deviation (SD). Intergroup differences were assessed with Student’s *t* test or analysis of covariances when adjusted for confounding factors, depending on the distribution normality of the tested parameter. *P* values <0.05 were considered statistically significant. The diagnostic value of each parameter was evaluated with the area under the receiving operator curve (ROC; AUC: area under the curve). Ninety-five percent confidence intervals of the AUC were calculated.

## Results

The final study population included 261 women, 87 with hip fracture and 174 controls without hip fracture. In the hip fracture group, 66.7% (58/87) had a *T*-score lower than −2.5 SD; in the control group, only 41.4% (72/174) had a *T*-score lower than −2.5 SD. Descriptive statistics of the study population are presented in Table 1.

**Table 1:** Characteristics of patients with fracture in comparison to those without fracture

	Subjects with fracture(*n* = 87)		Control subjects(*n* = 174)		*P* value
	Mean (range)	SD	Mean (range)	SD	
Age (years)	74.0(56~86)	6.6	73.9(56~85)	6.2	0.879
Height (cm)	157.1(143.8~175.0)	5.9	156.5(143.1~170.3)	4.6	0.371
Weight (kg)	60.8(36.1~80.9)	8.4	62.4(43.8~81.0)	8.0	0.147
BMI	24.7(11.9~35.6)	4.0	25.5(17.3~36.5)	3.6	0.103
Lumbar spine *T*-score	−2.2(−4.3~0.8)	1.1	−1.2(−3.3~2.2)	0.9	<0.001
Total hip *T*-score	−2.6(−4.9~1.3)	1.3	−1.6(−4.4~1.8)	1.4	<0.001
Femoral neck *T*-score	−2.7(−4.9~2.9)	1.4	−2.0(−4.4~1.8)	1.2	<0.001
Femoral trochanter *T*-score	−2.3(−5.1~0.6)	1.4	−1.6(−4.4~2.3)	1.3	<0.001
OSTA score	−2.2(−7~2)	1.7	−2.0(−6~3)	1.8	0.215
Singh index	2.9(1~6)	1.3	3.5(1~6)	1.2	<0.001

### Comparison between participants with and without fracture

There were no significant differences between the fracture and non-fracture groups in mean age (74.0 vs. 73.9 years, *P* = 0.879), height (157.1 vs. 156.5 cm, *P* = 0.897), weight (60.8 vs. 62.4 kg, *P* = 0.135), BMI (24.7 vs. 25.5 kg/cm^2^, *P* = 0.103), or OSTA scores (−2.3 vs. −2.0, *P* = 0.208). All BMD parameters at the lumbar spine and hip region (including total hip, femoral neck, and trochanter regions) were notably lower in the fracture group than in the non-fracture group, (−2.2 vs. −1.2, *P* < 0.0001 for lumbar spine; −2.6 vs. −1.6, *P* < 0.0001 for total hip; −2.7 vs. −2.0, *P* < 0.0001 for femoral neck; −2.3 vs. −1.6, *P* < 0.0001 for trochanter). In addition, SI scores were lower in the fracture group than in the non-fracture group (3.4 vs. 3.7, *P* = 0.017). All BMD parameters and SI scores remained significantly different when adjusted for BMI with covariance. Results are presented in Table 1.

### ROC curve

**Fig. 1:**
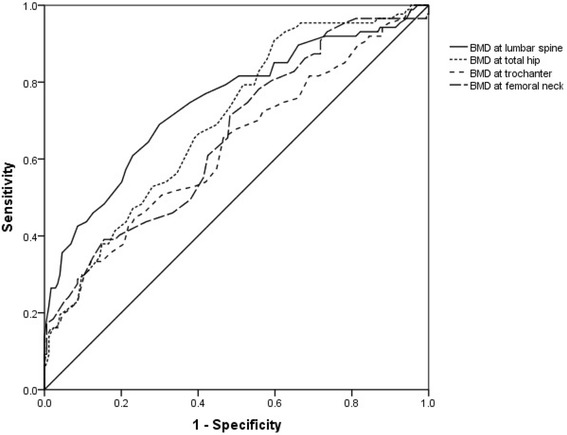
The ROC curves for BMD

With regard to hip fracture, the screening test performance characteristics and ROC curves are shown in Table 2 and Fig. 1. The AUC was 0.534 (95% CI: 0.459–0.610) for the OSTA, 0.636 (95% CI: 0.564–0.709) for the SI, 0.747 (95% CI: 0.680–0.813) for lumbar spine BMD, 0.699 (95% CI: 0.633–0.764) for total hip BMD, 0.659 (95% CI: 0.589–0.729) for femoral neck BMD, and 0.631 (95% CI: 0.557–0.704) for trochanter BMD. The cutoffs were −2.5, 2.5, −1.85, −2.45, −2.05, and −2.25, respectively. Furthermore, the optimal cutoff point as defined with the Youden index (sensitivity + specificity − 1) yielded the maximum value. AUCs of these parameters from high to low were BMD (lumbar spine), BMD (total hip), BMD (trochanter), SI, BMD (femoral neck), and OSTA. Next, the OSTA plus SI combination model was obtained using a logistic regression process. The AUC of the combination of OSTA plus SI was 0.795 (95% CI: 0.734–0.857). The combination of OSTA plus SI was compared with other screening methods. The AUC for combined OSTA plus SI was significantly different from that of OSTA alone (95% CI: 0.173–0.349, *Z* = 5.817, *P* < 0.0001), SI alone (95% CI: 0.086–0.232, *Z* = 4.254, *P* < 0.0001), BMD of total hip (95% CI: 0.017–0.177, *Z* = 2.368, *P* = 0.0179), BMD of trochanter (95% CI: 0.084–0.246, *Z* = 3.982, *P* = 0.0001), and BMD of femoral neck (95% CI: 0.086–0.232, *Z* = 4.254, *P* < 0.0001). However, the combination was not significantly superior to BMD of the lumbar spine (95% CI: −0.027–0.125, *Z* = 1.258, *P* = 0.2084).

**Table 2:** ROC analysis of diagnostic performance characteristics of BMD, OSTA, and SI

	AUC	95% CI	Cutoff value	Sensitivity (%)	Specificity (%)	Positive likelihood rate (LR+)	Negative likelihood rate (LR−)
Lumbar spine *T*-score	0.747	0.680~0.813	−1.85	60.9	77	2.38	0.49
Total hip *T*-score	0.699	0.633~0.764	−2.45	52.9	71.8	1.71	0.67
Femoral neck *T*-score	0.659	0.589~0.729	−2.05	74.7	47.1	1.40	0.49
Femoral trochanter *T*-score	0.631	0.557~0.704	−2.25	50.6	69.5	1.53	0.73
OSTA score	0.534	0.459~0.610	−2.5	44.8	73.8	1.24	0.86
Singh index	0.636	0.564~0.709	2.5	42.5	88.2	1.95	0.74

## Discussion

In this study, we evaluated the potential predictive value of the SI, OSTA, and of the combination of SI and OSTA in differentiating diabetic women with hip fracture from those without hip fracture. We found that the combination of SI plus OSTA and BMD of the lumbar spine performed better than SI alone, OSTA alone, BMD of the femoral neck, and BMD of the trochanter. The most noticeable result is that the combination of SI plus OSTA may be of clinical benefit when distinguishing between diabetic women with hip fracture versus without hip fracture. To be widely useful for a large population with T2DM, an assessment tool must be as simple as possible. The SI and OSTA are both simple, quick, and inexpensive risk assessment tools. The combination of SI plus OSTA could be especially useful in communities where DXA is costly and inaccessible.

BMD is widely used to determine bone mass and fracture risk in patients with risk factors for osteoporotic fractures. However, previous studies have reported conflicting data on BMD and the risk of osteoporosis in patients with T2DM [30]. In the current study, we showed that all BMD parameters were lower in patients with fracture than in those without fracture. The prevalence of osteoporosis was 66.7% in the fracture group and 41.4% in the control group, which also showed that osteoporosis was high in prevalence in the subjects with hip fracture. However, based on AUC analysis, BMD in the lumbar spine was superior to BMD in other regions in predicting hip fracture. BMD only explains 70 to 75% of the variance in bone strength; the macrogeometry of cortical bone and the microarchitecture of trabecular bone may be responsible for much of bone strength [31–35]. For a given BMD, diabetic bone is more fragile and has a higher risk of fracture than non-diabetic bone [36–38]. Some studies have indicated that material changes and structural abnormalities lead to increased bone fragility in patients with diabetes [39–41]. The World Health Organization’s Fracture Risk Assessment Tool, which is designed to predict the 10-year probability of osteoporotic fracture based on femoral neck BMD and other clinical risk factors, has been shown to underestimate fracture risk in diabetic patients. Hence, T2DM presents specific challenges for fracture risk assessment.

**Fig. 2:**
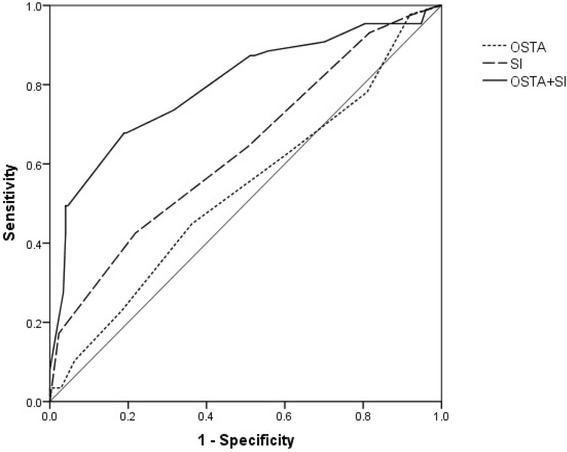
The ROC curves for OSTA, SI, and combination of OSTA plus SI

Because it is expensive and time consuming, DXA is not available at every healthcare center. The SI is a simple method of assessing bone strength that can be used to assess osteoporosis without DXA. Previous studies have shown that SI provides a reliable estimate of mechanical bone quality in addition to bone mass of the proximal femur [42–44]. In another study, Cemal et al. reported that the SI had relatively high specificity in predicting osteoporosis, but results were not consistent with those obtained with DXA because only bone loss beyond 30 to 50% can be present on plain radiographs [45]. In the present study, we adopted the Youden index to select an optimal cutoff threshold, which considered the balance between both sensitivity and specificity. The good performance is generally confirmed as AUC > 0.75. The best cutoff of OSTA and SI for predicting hip fracture were −2.5 and 2.5, respectively. It indicated further attention needed for high fracture risk. This study suggested that SI has a low predictive value (AUC 0.636) and sensitivity (42.5%), but good specificity (88.2%) in identifying hip fracture. SI alone was not an excellent predictive tool. Although the SI has shortcomings, it does have several advantages because it has good correlations with mechanical parameters of the trabecular bone of the proximal femur. In this study, we also evaluated the performance of the OSTA and found that the OSTA alone did not perform well in patients with diabetes. There was no significant difference in OSTA scores between the fracture group and control group. ROC analysis showed that the OSTA had a low predictive value (AUC 0.534, sensitivity 44.8%, specificity 73.8%, Fig. 2). Because the OSTA uses only age and weight as risk factors in its calculation, it can be distorted by obesity, which is widespread in T2DM. Overweight and obesity are generally believed to be protective factors for BMD through mechanical loading and hormonal factors, including insulin, estrogen, and leptin [46, 47]. Therefore, the OSTA index did not perform very well in identifying hip fracture in women with T2DM.

The present study provides further evidence about the combined use of the OSTA plus SI in assessing fracture risk in patients with T2DM. The data confirmed that the combination of the OSTA plus SI had a high predictive value (AUC 0.795), which was significantly higher than other screening tools. Combining these two assessments clearly enhanced their predictive ability. The findings highlight the importance of incorporating both OSTA and SI scores. However, the risk factors for hip fracture are complicated in patients with diabetes. For example, patients with T2DM are more likely to fall, but little is known about the specific risk factors for falling, including impaired visual acuity from diabetic retinopathy, balance disorders from coexisting sensory motor neuropathy, and diabetic foot [48]. On the other hand, T2MD may affect bone metabolism. As a result of concurrent albuminuria or exaggerated renal excretion of vitamin D metabolites, the vitamin D insufficiency has been linked to patients with diabetes compared to the general population [49]. Biochemical markers of bone metabolism, such as serum C-terminal telopeptide of type I collagen (β-CTX) and serum N-amino terminal prepeptide of type 1 procollagen (P1NP) have been found to be associated with skeletal health in T2MD [7, 30]. At the same time, there remained many controversies regarding the underlying mechanisms for bone fragility affected by diabetes metabolism. Therefore, an assessment tool for hip fracture risk must be further developed for the diabetic population.

The major limitation of this study is that was a retrospective case-control study. Prospective studies are more appropriate for the prediction of incident fractures. Our results cannot directly confirm an association between the value of various parameters and osteoporotic hip fracture. However, we believe that this limitation had little effect on the results. Second, the sample size was relatively small and without random control. These characteristics could have affected the spread of the data, which should be further confirmed in other larger cohorts. Furthermore, the OSTA was developed particularly in Asian countries. This tool may need to be validated in other ethnics.

## Conclusions

The combination of the OSTA plus SI could be useful for the clinical evaluation of hip fracture risk in women with T2DM, despite the paradox of fracture risk factors in this population. Considering that the OSTA and SI are the briefest and least expensive approaches to assessing bone health, a combination of the OSTA plus SI is suitable as a simple predictive tool in clinical practice to screen for hip fracture risk in a large diabetic population.

## Abbreviations

AUC: Area under the receiver operating characteristic curve
BMD: Bone mineral density
BMI: Body mass index
DXA: Dual-energy X-ray absorptiometry
OSTA: Osteoporosis Self-Assessment Tool for Asians
ROC: Receiving operator curve
SI: Singh Index
